# A Tactile Method for Rice Plant Recognition Based on Machine Learning

**DOI:** 10.3390/s20185135

**Published:** 2020-09-09

**Authors:** Xueshen Chen, Yuanyang Mao, Xu Ma, Long Qi

**Affiliations:** College of Engineering, South China Agricultural University, Guangzhou 510642, China; chenxs@scau.edu.cn (X.C.); mskobe@stu.scau.edu.cn (Y.M.); qilong@scau.edu.cn (L.Q.)

**Keywords:** rice, weeds, recognition, tactile, ANN

## Abstract

Accurate and real-time recognition of rice plants is the premise underlying the implementation of precise weed control. However, achieving desired results in paddy fields using the traditional visual method is difficult due to the occlusion of rice leaves and the interference of weeds. The objective of this study was to develop a novel rice plant recognition sensor based on a tactile method which acquires tactile information through physical touch. The tactile sensor would be mounted on the paddy field weeder to provide identification information for the actuator. First, a flexible gasbag filled with air was developed, where vibration features produced by tactile and sliding feedback were acquired when this apparatus touched rice plants or weeds, allowing the subtle vibration data with identification features to be reflected through the voltage value of an air-pressured sensor mounted inside the gasbag. Second, voltage data were preprocessed by three algorithms to optimize recognition features, including dimensional feature, dimensionless feature, and fractal dimension. The three types of features were used to train and test a neural network classifier. To maximize classification accuracy, an optimum set of features (b (variance), f (kurtosis), h (waveform factor), l (box dimension), and m (Hurst exponent)) were selected using a genetic algorithm. Finally, the feature-optimized classifier was trained, and the actual performances of the sensor at different contact positions were tested. Experimental results showed that the recognition rates of the end, middle, and root of the sensor were 90.67%, 98%, and 96% respectively. A tactile-based method with intelligence could produce high accuracy for rice plant recognition, as demonstrated in this study.

## 1. Introduction

Rice is one of the major global food crops and feeds over 65% of Chinese people [1]. One of the basic questions impeding the growth of crops concerns the competition of rice plants from weeds in farmland. Weeds in rice fields compete with rice for water, nutrients, and sunlight, resulting in a detrimental impact on rice yield and quality if not properly controlled [2].

Different operations have been attempted to control weeds, with chemical and mechanical weeding being widely used in rice fields nowadays. Conventional chemical weeding sprays herbicides uniformly to cover the total field, regardless of the presence of weeds or not, resulting in high herbicide costs. Overuse of herbicides in agriculture causes catastrophic environmental pollution problems, especially in China [3]. Another widely adopted weeding method is mechanical weeding, which is much more efficient but generally unsatisfactory in terms of weed control performance, causing differences in the bending of rice rows, leading to contact between weeding hoes and rice plants and potentially causing rice plant damage [4,5,6].

In this case, precise identification of rice plants is conducive to control weed growth, because it provides necessary information for subsequent decision-making and implementation procedures. Crop safety can be enhanced by adjusting the working path of weeding hoes or reducing the amount of herbicide applied through targeted spraying.

Many different sensing methods were attempted, within the critical period for weed control, an overall classification accuracy of 87 ± 5.57% was achieved for >5% vegetation coverage in a wheat field by field spectroscopy [7]. In the laboratory, under controlled illumination conditions, the hyperspectral imaging system provided high-quality images and high-accuracy differentiation of glyphosate-resistant (GR) weeds from glyphosate-sensitive (GS) weeds with accuracies from 75% to 95% [8]. In addition, visible and near infrared (Vis-NIR) spectroscopy [9], fluorescence [10], and distance sensing techniques (light detection and ranging-LiDAR and ultrasonic sensing) were also used for crop detection [11,12]. However, these methods are a non-real-time process and have poor anti-interference abilities in complex field environments.

Optical imaging using the machine vision technique is a very promising tool for precision farming and was investigated extensively for crop detection [13,14]. Using semi-supervised machine learning to recognize crops and weeds, Søgaard and Olsen [15] calculated the crop’s center of gravity in the horizontal direction to extract the navigation line. The critical procedure for precise rice detection is digital image processing, through which rice can be segmented and extracted from the acquired images. The recognition rate of the vision method for rice and weeds is only 85% [16], which is lower than the research method proposed in this paper. Image processing performance is dominantly influenced by complex paddy backgrounds, such as cyanobacteria or green algae, variable lighting conditions in the field, occlusion or overlapping of rice and weed leaves, different growth stages of plants, etc. [17,18,19].

In order to improve the anti-interference ability, several recent studies concerning crop classification by machine learning were designed. Cheng and Matson [20] used multiple machine learning algorithms such as decision tree, support vector machine (SVM) and neural network in rice and weed discrimination using images downloaded from the internet, achieving a best result of 98.2% precision. Hung et al. [21] classified three weed species using sparse autoencoders with precision scores of 72.2%, 92.9%, and 94.3% for each species. However, these methods using machine learning were of weaker performance during occlusion or overlapping of rice and weed leaves. Therefore, machine vision is more accurate in dry field crop recognition, but many interference factors exist when identifying rice plants or weeds in paddy field environments, therefore, the recognition process is not ideal.

Tactile methods provide an excellent solution to this problem because they do not depend on sunlight, background, and overlapping leaves. Wide varieties of tactile sensing technologies have been attempted, including optical [22], resistive [23], capacitive [24], piezoelectric [25], magnetic [26], and surface acoustic waves [27], among others, allowing the recognition of an object by gaining information such as contact shape, surface texture, roughness, and slippage detection [28,29,30,31,32,33], enabling tactile sensing to be of huge potential in areas of industry. Conversely, crop recognition based on tactile methods are used less in agricultural fields, unless there are significant characteristic differences between crops, such as physiological height or bending resistance. In the previous study, our laboratory team developed a rice recognition method based on a tactile method by using a bending sensor [34]. According to the difference of mechanical threshold between rice and weeds, the method could realize rice recognition in different water layer thickness and different rice varieties. However, rice recognition was highly dependent on the accuracy of mechanical threshold setting, which varied with the transplanting days, so the recognition rate was not stable. Xu Liming et al. [35] designed an auto-obstacle avoidance mechanism based on tactile perception for intra-row mechanical weeding between grape plants, with contact pressure detected by this machine when the contact rod was blocked by a grapevine. The automatic obstacle avoidance mechanism started to work when the contact pressure reached the threshold set by the control system. According to the differences in height and force between corn and weeds in the middle ploughing period, Jia Honglei et al. [36] designed a flexible shaft type tactile sensor to identify and locate corn crops by setting a reasonable contact position and contact force threshold. However, these tactile methods are unfit for identifying rice plants or weeds because the differences in height and contact force between rice plants and weeds are not as obvious as the stems of grapevines and corn. In addition, the mechanical recognition threshold of rice plants for different rice growing periods were constantly changing and were shown to not be significant. Further, the tactile feedback signal is complex and multidimensional, and cannot be directly used to obtain recognition information by setting an artificial fixed threshold.

Therefore, a rice plant recognition sensor which depended on pressure changes from a flexible gasbag when touching with rice plant or weeds based on machine tactile methods was proposed in this paper. To improve the recognition rate and anti-interference ability of the tactile sensor, it was used in the period when the differences in structure and mechanics between weeds and rice were significant (16–21 days after rice plants transplanting). During operation, the sensor moved along with the machine and its height was adjusted to ensure that the gasbag touched the rice stem and the canopy of weed. The tactile signals were obtained by sensor for deep mining. After conversion of low-level raw data into high-level information (feature extraction), the machine recognition of rice plant in weeds was realized by a back propagation (BP) neural network. In this paper, the main purpose of this study was to identify rice plants among weeds using a tactile method. One key technology involved the acquisition of subtle data with identifying features for rice plants and weeds. Another was the effective classification of the extracted sensing data.

Our contributions are mainly as follows.

1. A novel sensing method for identifying rice plants and weeds was proposed to address the poor effect of the vision recognition method in rice field, which was different from the previous tactile perception method based on artificial threshold recognition. This study was based on the features of tactile perception data of rice plants and weeds.
2. A flexible tactile sensor was designed. The gasbag structure of the cantilever beam type showed good adaptability and barometric sensitivity, which was conducive to obtaining differences in structure and mechanics between rice plants and weeds and provided a basis for the depth mining of tactile identification data of rice plants and weeds.
3. A classification method of rice plants and weeds was proposed, including feature extraction with dimension, dimensionless, and fractal dimension, feature selection with genetic algorithm, and feature classification with neural network. To some extent, this improved the accuracy of identification of rice plants and weeds.

## 2. Materials and Methods

### 2.1. Tactile Signals Acquisition and Processing

**Figure 1:**
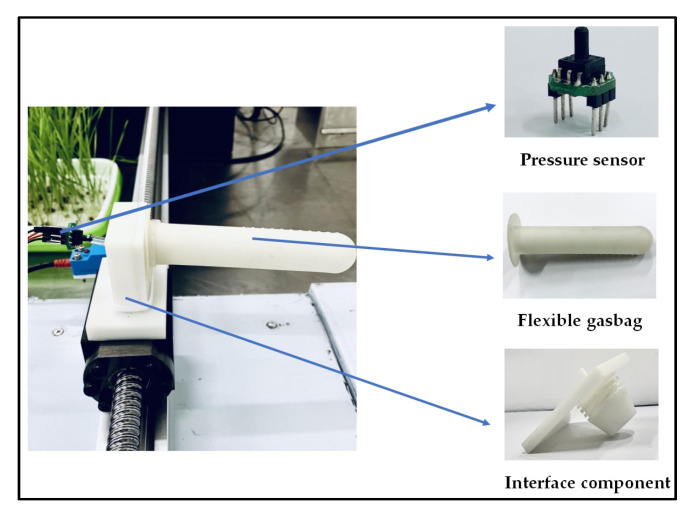
Diagram of rice plant recognition sensor.

**Figure 2:**
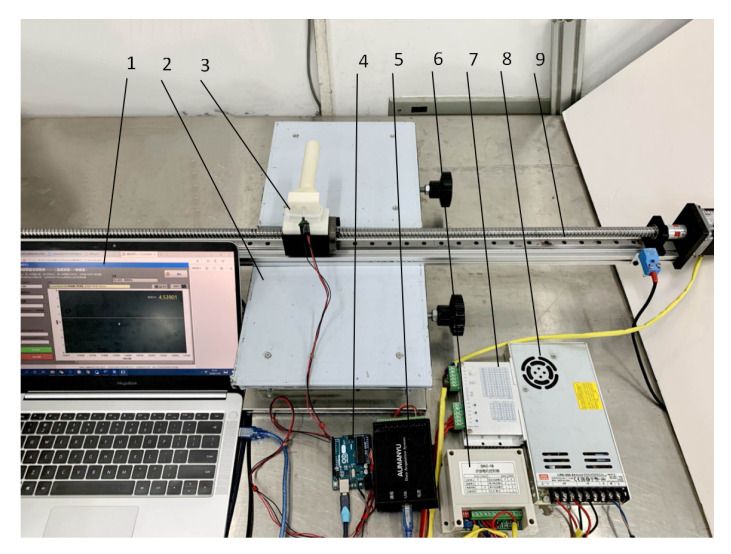
Diagram of experimental device: (**1**) master computer program, (**2**) lifting platform, (**3**) flexible gasbag with air pressure measurement, (**4**) Arduino, (**5**) 24-bit AD data acquisition module (USB DAQ-580I), (**6**) controller, (**7**) driver, (**8**) DC power, and (**9**) line-glide rail.

To acquire the tactile signals for recognizing rice plants among weeds, a rice plant recognition sensor was designed (Figure 1), allowing the production of voltage signals with identifying features as it was bent by rice plants or weeds. The rice plant recognition sensor was composed of two main components, i.e., the flexible gasbag and the pressure sensor. The flexible gasbag (Jinyi 3D Printing Technology Co., Ltd., Dongguan, China) was made of rubber, allowing it fully squeeze alongside rice plants or weeds to acquire the microvibration identifying characteristics in the process of contact friction. The air pressure in the gasbag was atmospheric pressure. The connection between the pressure sensor and the flexible gasbag is shown in Figure 1. The pressure sensor (XGZP6847, Anhui core silicon Intelligence Electronic Technology Co., Ltd., Wuhu, China), which was designed for accurate pressure measurements, was used in the rice plant recognition sensor. The measured range of pressure sensor varied from −100 to 1000 kPa. The detected data was collected by Arduino (Longzhan Information Technology Co., Ltd., Shanghai, China), which transmitted the data to the 24-bit analog to digital (AD) data acquisition module (USB DAQ-580I, Oumanyu Intelligent Technology Co., Ltd., Suzhou, China) through the serial port. The master computer program was programmed using the laptop to read and process the data and control the beginning and end of the collecting time. The rice plant recognition sensor was installed horizontally on a line-glide rail, including a DC power supply, a controller, and a driver (Figure 2). Supplied by DC power, the controller and driver made the line-glide rail work according to the preset path.

**Figure 3:**
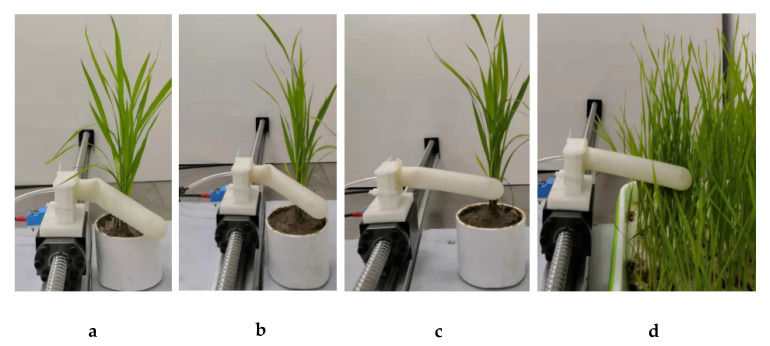
Contact process of gasbag with rice and weeds: (**a**) contact between the root of the gasbag and rice; (**b**) contact between the middle of the gasbag and rice; (**c**) contact between the end of the gasbag and rice; (**d**) contact between the gasbag and weeds.

The contact position between weeds and the gasbag was not clear because the weeds grow in clusters and the stems were scattered. In contrast, rice grows as a hole planting crop, and the stem base of rice is concentrated. The contact position with the gasbag can be clearly divided into three situations: the root, the middle and the end of the gasbag (Figure 3). When the flexible sensing gasbag touched the rice stem or the canopy of weed, the gasbag formed a local deformation at the contact position. In the process of mutual contact sliding, a series of microvibrations occurred, resulting in regular changes in internal air pressure, with pressure change data (tactile voltage signal) obtained through the pressure sensor. The length of each acquired tactile voltage signal was kept the same using the master computer program to control the beginning and end of the AD data acquisition module. 

To process the data, the master computer program stored the tactile voltage signals from the AD data acquisition module as a text document in the format of TXT. R2018a (version 9.5) MATLAB software was used to import the tactile voltage signals for feature extraction. Then, the extracted features were imported into Python (version 3.7 64-bit) and a genetic algorithm programs were written in Python (version 3.7 64-bit), which was used for feature selection. Five times feature selections were made to determine the identification features of rice plants and weeds. Next, a rice plant and weed classifier based on a BP neural network was constructed using R2018a (version 9.5) MATLAB software. The above features were selected to form a feature vector, and some of the feature vector samples were used as training sets to input the classifier, with other samples used as testing sets.

### 2.2. Plant Growth Conditions

The rice samples were taken from rice transplant fields at South China Agricultural University in May 2019. Each sample was placed in a plastic container. The morphology of gramineous weeds was similar to rice plants, so it was difficult to distinguish between gramineous weeds and rice plants using the visual method. In this paper, gramineous weeds were selected as samples of weeds. The samples were kept under controlled conditions in a greenhouse (temperature between 23 and 27 °C and a relative humidity of 80% ± 15%). All rice plant and weed samples were used for tactile signal acquisition in one day. Figure 3 depicts the laboratory experiment.

### 2.3. Data Processing

#### 2.3.1. Feature Extraction

Dimensional parameters and dimensionless parameters were used in numerous research studies to measure signal characteristics [37,38]. Fractal theory effectively describes the irregularity of tactile voltage signals, revealing that local signals show similarity with whole signals in a certain aspect [39]. The fractal dimension of the signals reflects the complexity of the signal geometry. In this research, 5 dimensional parameters, 6 dimensionless parameters, and 2 fractal dimension features were extracted from the tactile voltage signals of rice and weeds and used to train the classifier. Furthermore, the tactile signals were extracted through three positions of the flexible gasbag to determine whether there were significant differences between the features (dimensional parameters, dimensionless parameters, and fractal dimension features) of the tactile signals from rice plants and weeds.

##### Dimensional Parameter

The dimensional parameter feature set included the mean value, variance, standard deviation, root mean square, and peak-to-peak value. The dimensional parameters were obtained from Equations (1)–(5), respectively.

(1) $$\mu_{x}\left(t\right)=\frac{1}{n}\overset{n}{\underset{i=1}{\sum }}x_{i}\left(t\right)$$

(2) $$\sigma_{x}^{2}\left(t\right)=\frac{1}{n}\overset{n}{\underset{i=1}{\sum }}{\left[x_{i}\left(t\right)-\mu_{x}\left(t\right)\right]}^{2}$$

(3) $$\delta_{x}\left(t\right)=\sqrt{\frac{1}{n}\overset{n}{\underset{i=1}{\sum }}{\left[x_{i}\left(t\right)-\mu_{x}\left(t\right)\right]}^{2}}$$

(4) $$RMS=\sqrt{\frac{1}{n}\overset{n}{\underset{i=1}{\sum }}x_{i}^{2}\left(t\right)}$$

(5) PK=max (x(t))−min(x(t))

where x is the amplitude of tactile voltage signal, n is the number of sampling points for the tactile signal, $\mu_{x}\left(t\right)$ is the mean value, $\sigma_{x}^{2}\left(t\right)$ is variance, $\delta_{x}\left(t\right)$ is standard deviation, RMS is root mean square, PK is peak-to-peak value.

##### Dimensionless Parameter

The dimensionless parameter feature set included kurtosis, skewness, waveform factor, pulse factor, peak factor, and margin factor. Kurtosis, skewness, and margin factor were calculated directly by the function of R2018a (version 9.5) in the MATLAB software toolbox. Waveform factor, pulse factor, and peak factor were obtained from Equations (6)–(8), respectively.

(6) $$S=\frac{RMS}{\left|\mu_{x}\left(t\right)\right|}$$

(7) $$C=\frac{\max\;\left(x\left(t\right)\right)}{\left|\mu_{x}\left(t\right)\right|}$$

(8) $$I=\frac{\max\;\left(x\left(t\right)\right)}{RMS}$$

where x is the amplitude of tactile voltage signal, n is the number of sampling points for the tactile signal.

##### Fractal Dimension Feature

The fractal dimension is a main measurement tool used in signal processing technologies. Fractal-based feature extraction measures the change in the distribution of signal complexity. Box dimension and the Hurst exponent were selected in this paper because they were easy to calculate and have anti-noise ability [39], and they were obtained from Equations (9) and (10), respectively [40].

Box dimension was presented by Bouligand (1929). Box dimension is easy to calculate and measure. Let F be any nonempty bounded subset of $R_{n}$, N(X,δ) is the minimum number of sets that can cover F, and the maximum diameter is δ, therefore, the box dimension of F is obtained by:

(9) $$F_{B}=\;\underset{\delta \to 0}{\lim}\frac{\ln N\left(F,\delta \right)}{\ln\left(\frac{1}{\delta }\right)}.$$

The Hurst exponent is a dimensionless estimate of self-similarity measure, which is usually used to represent the correlation of time series in a long range. Hurst exponent can be obtained by:

(10) $$\log_{10}\frac{R\left(N\right)}{S\left(N\right)}=H\log_{10}N+C$$

where C is a constant and R/S is the rescaled range. $\log_{10}\frac{R\left(N\right)}{S\left(N\right)}$ and $\log_{10}N$ are regressed by the least square method. The slope of the regression line is the estimated value of Hurst exponent.

#### 2.3.2. Feature Selection

Proper feature selection can increase the performance of an inference model, the genetic algorithm (GA) can find optimal numbers for several diverse features and improve classification accuracy by selecting appropriate features and removing less important ones [41], so a GA was applied to select the features. In this research, crossover with mutation was utilized and operated on a population of binary-encoded chromosomes, with each chromosome representing n candidate features [41]. The parameters of genetic algorithm include crossover probability, mutation probability and population size, which were set to 0.8, 0.01 and 500 respectively. Separability criterion based on the distance between classes was used for the selection of parent chromosomes for the next generation. The fitness function in this research was obtained through Equations (11)–(15) [42].

(11) $$J_{d}\left(x\right)=\;\frac{1}{2}\overset{c}{\underset{i=1}{\sum }}P_{i}\overset{c}{\underset{j=1}{\sum }}P_{j}\frac{1}{n_{i}n_{j}}\overset{n_{i}}{\underset{k=1}{\sum }}\overset{n_{j}}{\underset{l=1}{\sum }}\delta \left(x_{k}^{\left(i\right)},x_{l}^{\left(j\right)}\right)$$

(12) $$\delta \left(x_{k}^{\left(i\right)}+x_{l}^{\left(j\right)}\right)=\;{\left(x_{k}^{\left(i\right)}-x_{l}^{\left(j\right)}\right)}^{T}\left(x_{k}^{\left(i\right)}-x_{l}^{\left(j\right)}\right)$$

(13) $$m_{i}=\;\;\frac{1}{n_{i}}\overset{n_{i}}{\underset{k=1}{\sum }}x_{k}^{\left(i\right)}$$

(14) $$m=\;\overset{c}{\underset{i=1}{\sum }}P_{i}m_{i}$$

(15) $$Fitness\;Function=\;\overset{c}{\underset{i=1}{\sum }}P_{i}\left[\frac{1}{n_{i}}\overset{n_{i}}{\underset{k=1}{\sum }}{\left(x_{k}^{\left(i\right)}-m_{i}\right)}^{T}\left(x_{k}^{\left(i\right)}-m_{i}\right)+{\left(m_{i}-m\right)}^{T}\left(m_{i}-m\right)\right]$$

where $J_{d}\left(x\right)$ is the average distance between various features, $\delta \left(x_{k}^{\left(i\right)}+x_{l}^{\left(j\right)}\right)$ is Euclidean distance, $m_{i}$ is mean vector of class i sample set, m is the total average vector of all sample sets. The weeds are regarded as class *W_i_* and rice as class *W_j_*. *d* features were selected to optimize the criterion function from *D* features (*d* < *D*). *n_i_* and *n_j_* are the number of samples of class *W_i_* and *W_j_* respectively, *P_i_* and *P_j_* are the corresponding prior probabilities, *c* is the number of categories, and $x_{k}^{\left(i\right)}$ and $x_{l}^{\left(j\right)}$ are *D*-dimensional vectors in class $W_{i}$ and class $W_{j}$, respectively.

By applying the fitness function, the primary population (initial subset) was selected. If the selected subset satisfied the predefined criterion, this was reported as the optimal feature subset. Otherwise, a new feature subset was selected using two important genetic operators called the crossover and mutation operators. Then, this new subset was selected again using the fitness function. After selection, crossover, and mutation, the initial subset became a new generation. The above process was repeated iteratively, with the chromosome in the population tending toward the optimal solution of the selected characteristic number, allowing the algorithm to stop. The implementation of the genetic algorithm was conducted in Python (version 3.7 64-bit).

#### 2.3.3. Data Classification

The artificial neural network was used to separate and classify the feature dataset 41]. The BP neural network has no strict requirements for the data distribution, it can automatically transform the initial “bottom” feature representation into a “high-level” feature through a multilevel and nonlinear transformation [43], which ensured that rice and weeds were effectively identified with distinctive parameters. In this study, a three-layer BP neural network (input layer, hidden layer and output layer) was designed to construct a classifier, which can accurately realize any continuous mapping. According to the Kolmogorov theory [44], the number of neurons in the hidden layer met the condition: D ≥ 2M + 1, where D is the number of neurons in the hidden layer and M is the number of input nerves. The number of neural nodes of the input layer corresponded to the features. The number of neural nodes of the output layer corresponded to the weeds and rice. The number of neurons in the hidden layer was 12, which was confirmed by testing. The training function of the BP neural network was a gradient descending function based on the adaptive learning rate. The learning algorithm of the connection weights and the threshold values was a momentum-learning algorithm based on gradient descending. For training and testing classifier, data were first randomly split into two parts, with 1200 data selected for train the network and 600 data selected to test the network.

### 2.4. Experimental Methods

There were three possible cases for an experiment in practice. To verify the accuracy of the sensor in recognizing rice plants under the interference of weeds, the following experiment was carried out. In this experiment, three cases were carried on and compared, and the average of the recognition accuracy was calculated.

Case I: Touching rice plants and weeds with the end of gasbag while moving the sensor.
Case II: Touching rice plants and weeds with the middle of gasbag while moving the sensor.
Case III: Touching rice plants and weeds with the root of gasbag while moving the sensor.

The number of samples of rice plants in each experiment was 150, and the number of rice plants recognized correctly was counted to calculate the recognition rate.

In the experiment, the field environment was used as a reference standard. Rice plants were transplanted for 16–21 days and weeds corresponding to the number of days of rice plant were collected. During the experiment, weeds were placed in two 0.3 × 0.5 m trays with a density of 1-plant per square centimeter. Rice plants placed in plastic containers were arranged into a row on trays every 15 cm (5 rice plants were placed each time, and the operation was repeated thirty times). The speed of the sensor was 1-m per second, and the contact height between gasbag and plants was 25 cm (stem base of the rice plant, middle of the stem of the weed, and canopy of the weed).

## 3. Results

### 3.1. Comparison of Tactile Signals

**Figure 4:**
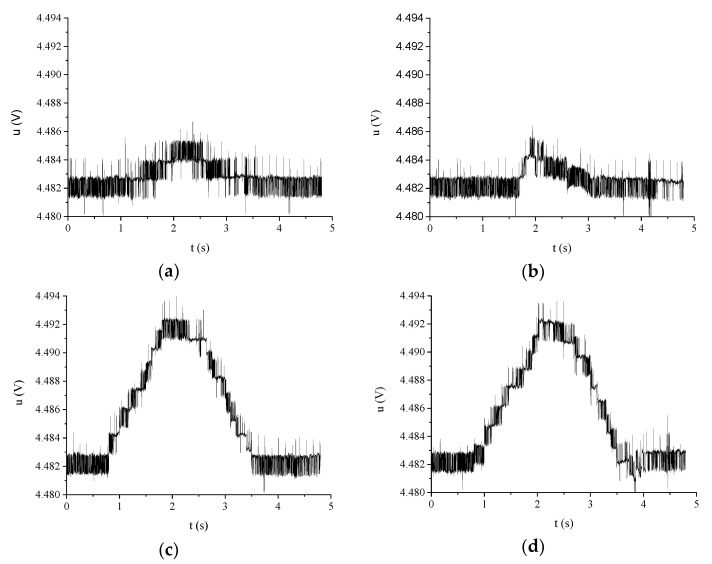
Waveforms of tactile voltage signals: (**a**) contact between the gasbag and weeds; (**b**) contact between the gasbag end and rice; (**c**) contact between the middle of the gasbag and rice; and (**d**) contact between the root of the gasbag end and rice.

Under different contact positions, the voltage of tactile signals fluctuated consequently, with extracted features also changing. The tactile voltage signals of rice and weeds were plotted into waveforms (Figure 4). Figure 4c represents a waveform diagram which was generated when the middle part of the gasbag touched the rice (Type C), and Figure 4d is a waveform diagram that was generated when the root part of the gasbag touched the rice (Type D). It can be clearly seen that the amplitudes of these two waveforms were significantly larger than the waveform of the gasbag in contact with the weeds (Figure 4d). Using the peak-to-peak value, rice and weeds were easily distinguished. Figure 4b shows a waveform diagram of the contact between the gasbag end and rice (Type B), with Figure 4a showing a waveform diagram of the contact between the gasbag and weeds (Type A). Figure 4c is very similar to Figure 4d. It was difficult to distinguish between rice and weeds using a certain feature, therefore, it was necessary to use multiple features of different types in combination with machine learning to distinguish between rice and weeds.

### 3.2. Feature Extraction Results

Table 1, Table 2 and Table 3, which present the dimensional parameter, dimensionless parameter, and fractal dimension feature extracted from the tactile signals (1800 data), respectively, show no clear trend. For this reason, feature selection was used for classification.

### 3.3. Results of The Tested Network Accuracy for Each Group

Based on the results of the feature extraction, the extracted parameters from tactile signals were categorized into three groups, namely, Group I for dimensional parameters, Group II for dimensionless parameters, and Group III for fractal dimension features.

Three categories of features from the tactile signals were used as neurons in the input layer. A total of 1200 data were selected to train the network and 600 data were selected to test the network. According to previous scholars’ processing methods of neural network on testing sets [45], if only one test set was selected, the accuracy of the test set would fluctuate due to the different recognition accuracy of the neural network for each type. Therefore, the performance of the classifier can be better evaluated by dividing the testing set into three groups, with 200 data in each group. The numbers of weeds and rice in each dataset are shown in Table 4. The results of the network accuracy for each group are shown in Table 5, Table 6 and Table 7. It can be seen from the tables that the three types of features were used as the input layer of the neural network to train the neural network. The accuracy was low and showed no clear statistical pattern, making it difficult to identify the weeds and rice. Therefore, a genetic algorithm was applied to select the features. The GA found optimal numbers for several diverse features and improved the classification accuracy by selecting appropriate features and removing less important ones.

### 3.4. Feature Selection and Performance of Classifiers

**Figure 5:**
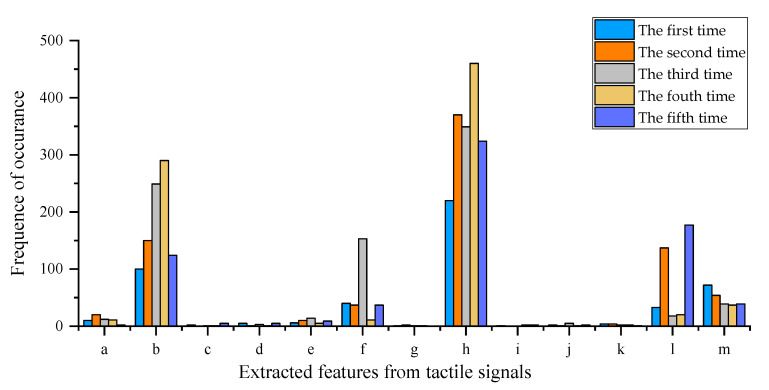
Resulting plot of five times feature selection using the genetic algorithm.

Features extracted from the tactile signals included the mean value (a), variance (b), standard deviation (c), root mean square (d), peak-to-peak value (e), kurtosis (f), skewness (g), waveform factor (h), pulse factor (i), peak factor (j), margin factor (k), box dimension (l), and Hurst exponent (m). Based on the grouping of the extracted parameters as described above, the GA was used in 500 iterations to select the most appropriate features. The frequencies of occurrence for each feature after five times feature selection are shown in Table 8 and Figure 5. In five times feature selection, variance, kurtosis, waveform factor, box dimension, and the Hurst exponent showed great advantages in 500 genetic iterations. The parameters with the largest number of occurrences were considered to be the most appropriate features (Group IV) to train the BP neural network.

**Figure 6:**
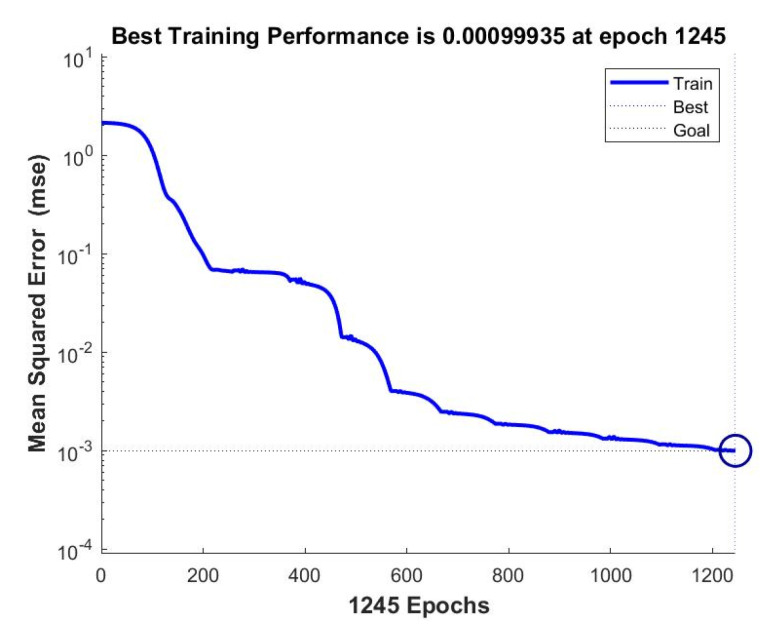
The training result of the back propagation (BP) neural network.

The training curve denoting the training procedure is shown in Figure 6. The curve was convergent when the training goal, describing the error between the training output and the ideal output, was defined as $10^{-3}$. The goal was reached at 1245 epochs in this training procedure. Therefore, the result of the training procedure was satisfactory.

**Figure 7:**
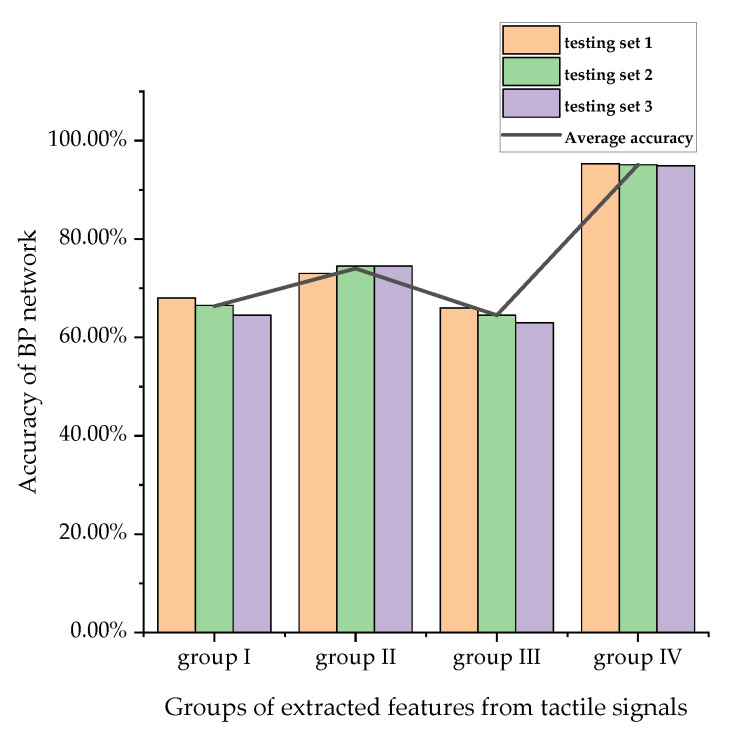
Resulting plot of the comparison of recognition rate before and after using the genetic algorithm.

Table 9 demonstrates the performance of the BP neural network classifier. According to this table (Table 9), the best accuracy of the classifier for each testing set was observed. The rice recognition rates of the classifier for the three testing sets were 95.3%, 95.1%, and 94.9%. This result was satisfactory, which greatly improved the accuracy compared with the result without using the genetic algorithm (Figure 7). Therefore, the trained classifier could be used for rice and weeds recognition and classification.

### 3.5. Performance of Rice Machine Recognition Sensor

**Figure 8:**
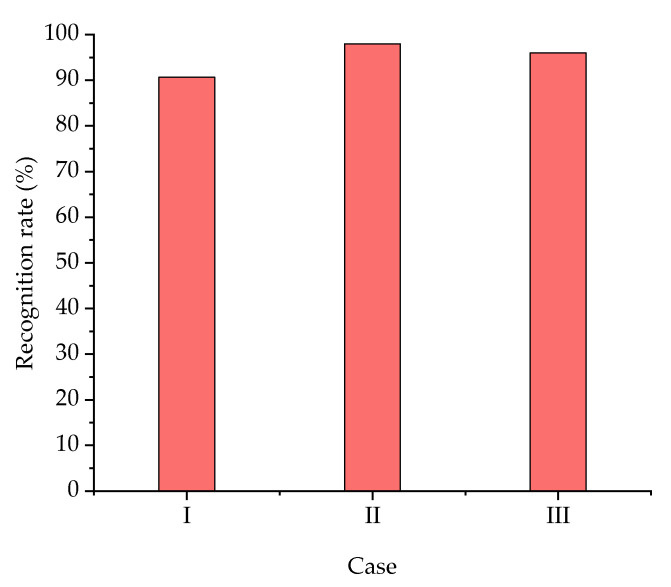
Resulting plot of the experiment.

The recognition results of the experiment were 90.67%, 98%, and 96%, respectively (Figure 8). The average recognition rate of the experiment was 94.89%.

## 4. Discussion

According to the results of case I, the recognition rate of rice plants was high, at 90.67%. However, it was difficult to produce slipping characteristics when rice plants were touched using the extreme end of gasbag. Therefore, the recognition rate was influenced due to less obvious slipping characteristics.

According to the results of case II, the recognition rate of rice plants was as high as 98%, representing the highest rate among the three cases (Figure 8) and meaning that the recognition of rice plants using this method was reasonably good. The high recognition rate was due to the obvious slipping characteristics produced by the middle part of gasbag touching the rice plants.

According to the results of case III, the recognition rate of rice plants was slightly lower than in case II, at 96%. There were two situations that caused recognition error related to the process of the gasbag touching the rice plants. The first involves the extreme root of the gasbag touching the rice plants, and the second is the gasbag touching weak rice plants (with a tiller number of less than three). In these situations, the bending stiffness of the root of gasbag was large due to the cantilever structure with no obvious deformation of the gasbag, resulting in a lack of obvious slipping characteristics. Therefore, recognition error occurred.

In summary, the average recognition rate of the experiment was 94.89%, which was relatively high due to the obvious mechanical identification properties of rice plants with long transplantation periods; the recognition rate was relatively high when there were obvious slipping characteristics of mutual friction (i.e., the middle of the gasbag touched the rice plants). Therefore, parameters such as the length of the gasbag should be optimized according to the rice planting pattern. In this paper, the three categories of features selected for neural network are commonly used as signal analysis features [36,37,38]. The results showed that the accuracy of the proposed sensor for rice recognition was satisfactory. However, the sensor needed a certain sliding friction with the rice plant to achieve more accurate identification. Therefore, the recognition had a short delay which should be considered in the operation of guiding weeding parts or herbicide sprinklers to avoid rice plants. In addition, the sensor was mainly used for the recognition of rice plants during the period of weed control, and the recognition error was allowed to be increased in the late stage of rice growth when the mechanical and height differences between rice plants and weeds were not obvious. It should be mentioned that, in the process of feature selection using genetic algorithm, some features may not be selected, which reduces the accuracy of selecting the best features to some extent. Therefore, more features selection methods need to be carried out to optimize features. More recognition models such as decision tree and support vector machine should be compared, and the best recognition model can be used by comparing the recognition results. In future studies, the parameters that can better reflect the tactile signal characteristics should be introduced to further improve the recognition accuracy of sensor. In addition, appropriate transplanting periods should be selected according to the agronomy of rice plant growth (bending strength of the stem). The sensor should be waterproofed in the future to facilitate field measurement.

## 5. Conclusions

In this study, a rice plant recognition sensor was developed using a tactile method and machine learning algorithm. Tactile information was acquired from voltage signals of an air-pressure sensor in a gasbag which touched rice plants. During data processing, three algorithms were used to extract 13 features of tactile voltage signals, and an optimum set of features (variance, kurtosis, waveform factor, box dimension, and hurst exponent) was selected using a genetic algorithm. A rice plant and weed classifier was built using a BP neural network. The rice recognition rates for the three testing sets were 95.3%, 95.1%, and 94.9%.

Based on the proposed classifier, an experiment with three case was designed according to the different positions of the gasbag touching the rice plants and weeds. The best recognition performance was achieved by the middle of gasbag touching the rice plants, with the recognition rate being as high as 98%. The second-best recognition performance was achieved by the root of gasbag touching the rice plants, at 96%. When the end of the gasbag touched the rice plants, the recognition rate was the lowest that was observed in the experiment, at 90.67%. The dataset in this paper were obtained from a single rice variety, so the data of the corresponding varieties need to be obtained to train the classifier for the recognition of other rice varieties. The experiment proved that tactile-based recognition of rice plants is a promising method.

## Figures and Tables

**Table 1:** Mean comparison of the dimensional parameter from tactile signals.

Type	Mean Value	Variance	Standard Deviation	Root Mean Square	Peak-to-Peak Value
A	4.4887085163	0.0000010871	0.0010409263	4.4897086375	0.0065800754
B	4.4901966346	0.0000011615	0.0009045177	4.4901967198	0.0070144666
C	4.5020162453	0.0000130044	0.0036061615	4.4941781713	0.0137633645
D	4.5016362546	0.0000125733	0.0035458856	4.4937001083	0.0136222347

**Table 2:** Mean comparison of the dimensionless parameter from tactile signals.

Type	Kurtosis	Skewness	Waveform Factor	PULSE FACTOR	Peak Factor	Margin Factor
A	2.9811741532	0.5894722756	1.0000000269	0.0014659157	0.0014659156	0.0014659157
B	4.5065444387	0.6108866395	1.0000000312	0.0015621864	0.0015621865	0.0015621864
C	1.6274810909	0.2772723874	1.0000003219	0.0030624074	0.0030624064	0.0030624079
D	1.8685779661	0.4941003920	1.0000003113	0.0030313559	0.0030313549	0.0030313564

**Table 3:** Mean comparison of the fractal dimension from tactile signals.

Type	Box Dimension	Hurst Exponent
A	1.5932511530	0.9627329822
B	1.5657002652	0.9406033958
C	1.5636990305	1.0030099281
D	1.5647215164	1.0028526065

**Table 4:** Data allocation of training set and testing sets.

Type	Training Set	Testing Set 1	Testing Set 2	Testing Set 3
A	300	50	57	43
B	300	50	43	57
C	300	50	50	50
D	300	50	50	50
Total	1200	200	200	200

**Table 5:** Network accuracy results for group I.

Type	Testing Set 1	Testing Set 2	Testing Set 3
Correct number of type A	40	43	35
Correct number of type B	28	26	33
Correct number of type C	40	39	40
Correct number of type D	39	36	37
Accuracy of weeds	80%	75.4%	81.4%
Accuracy of rice	71.3%	70.6%	70.1%

**Table 6:** Network accuracy results for group II.

Type	Testing Set 1	Testing Set 2	Testing Set 3
Correct number of type A	38	45	33
Correct number of type B	35	31	41
Correct number of type C	38	39	37
Correct number of type D	35	34	38
Accuracy of weeds	76%	78.9%	76.7%
Accuracy of rice	72%	72.7%	73.9%

**Table 7:** Network accuracy results for group III.

Type	Testing Set 1	Testing Set 2	Testing Set 3
Correct number of type A	39	43	32
Correct number of type B	40	33	44
Correct number of type C	40	41	41
Correct number of type D	42	43	41
Accuracy of weeds	78%	75.4%	74.4%
Accuracy of rice	81.3%	81.8%	80.3%

**Table 8:** Results of five times feature selection using the genetic algorithm.

Type	The First Time	The Second Time	The Third Time	The Fourth Time	The Fifth Time
a	10	20	12	11	2
b	100	150	249	290	124
c	2	0	1	1	5
d	5	1	3	1	5
e	6	10	14	5	9
f	40	37	153	11	37
g	1	2	1	1	0
h	220	370	349	460	324
i	1	0	0	2	2
j	2	1	5	1	2
k	4	4	2	2	1
l	33	137	18	20	177
m	72	54	39	37	39

**Table 9:** Network accuracy results for group IV.

Type	Testing Set 1	Testing Set 2	Testing Set 3
Correct number of type A	41	46	35
Correct number of type B	46	39	52
Correct number of type C	49	50	49
Correct number of type D	48	47	48
Accuracy of weeds	82%	80.7%	81.4%
Accuracy of rice	95.3%	95.1%	94.9%
